# Prevalence of Multidrug-Resistant Bacteria in Healthcare-Associated Bloodstream Infections at Hospitals in Riyadh, Saudi Arabia

**DOI:** 10.3390/pathogens12091075

**Published:** 2023-08-23

**Authors:** Saeed S. Banawas, Ahmed S. Alobaidi, Turki M. Dawoud, Abdullah AlDehaimi, Faisal M. Alsubaie, Ahmed Abdel-Hadi, Palanisamy Manikandan

**Affiliations:** 1 Department of Medical Laboratories, College of Applied Medical Science, Majmaah University, Al-Majmaah 11952, Saudi Arabia; fm.alsubaie@mu.edu.sa (F.M.A.); a.abdelhadi@mu.edu.sa (A.A.-H.); m.palanisamy@mu.edu.sa (P.M.); 2 Health and Basic Sciences Research Center, Majmaah University, Al-Majmaah 11952, Saudi Arabia; 3 Department of Biomedical Sciences, Oregon State University, Corvallis, OR 97331, USA; 4 Department of Botany and Microbiology, College of Science, King Saud University, P.O. Box 4255, Riyadh 11451, Saudi Arabia; ahalobaidi@ksu.edu.sa (A.S.A.); tdawoud@ksu.edu.sa (T.M.D.); 5 MBS-Infection Control, King Fahad Medical City, Riyadh 12211, Saudi Arabia; aldehaimi.a10@gmail.com

**Keywords:** bloodstream infections, multidrug-resistant bacteria, gram-negative bacteria, gram-positive bacteria, healthcare

## Abstract

Bloodstream infection (BSI) prevalence in hospitalized patients has increased owing to the spread of antibiotic-resistant pathogens; moreover, antimicrobial resistance in bacteria is a global problem. Here, BSIs are investigated in several patients at a hospital in Saudi Arabia, and the resistance of bacterial isolates to widely used drugs is determined. Throughout 2020, bacteria isolated from patients were identified and subjected to antibiotic susceptibility testing. In total, 1125 bacterial isolates were obtained from 1039 patients; among them, gram-positive bacteria were significantly more abundant than gram-negative bacteria. The most prevalent bacteria were *Staphylococcus epidermidis* and *Klebsiella pneumoniae*. Notably, gram-negative bacteria were mainly isolated from adult patients, and 20.63% of the gram-positive isolates were from pediatric patients, which was significantly higher than the corresponding percentages in elders and adults. The gram-positive isolates were mainly resistant to cephalothin, oxacillin, amoxicillin-clavulanate, and erythromycin and susceptible to penicillin, gentamicin, ciprofloxacin, and vancomycin. Additionally, the gram-negative isolates were mainly resistant to ampicillin, cephalothin, and amoxicillin-clavulanate and susceptible to amikacin, ertapenem, aztreonam, colistin, and trimethoprim-sulfamethoxazole. Consequently, the high prevalence of infective multidrug-resistant bacteria may account as a significant health issue; it is considered a hazard in Riyadh hospitals and must be prevented at all costs.

## 1. Introduction

Bloodstream infections (BSIs) are characterized by the presence of live bacteria or fungi that trigger inflammatory reactions [1]. Tests based on clinical, laboratory, and hemodynamic parameters can be used to identify certain BSIs. Both BSIs and sepsis are similar in terms of being infectious diseases, and BSIs may be considered sepses because they involve the circulation of live bacteria in the blood [1]. BSIs are a major cause of morbidity and mortality worldwide due to infectious diseases. Sepsis is also a significant cause of neonatal morbidity and death despite recent improvements in healthcare. In addition, sepsis is often associated with substantial morbidity and mortality after the transplantation of hematopoietic stem cells [2,3]. Although end-stage liver disease is one of the most commonly acquired infections in the intensive care unit (ICU), the highest mortality rates among hospitalized patients have been associated with BSI-resistant microorganisms [4,5]. Globally, nearly 49 million people are infected with BSIs annually, resulting in approximately 11 million deaths. Moreover, every year, three million babies and one million patients suffer from sepsis [6,7]. Between 1981 and 2015, the proportion of BSIs in Saudi Arabia ranged from 1% to 11% in patients with confirmed infections [8]. In addition, among the Gulf Cooperation Council hospitals, the risk of acquiring central line-associated BSIs was 146% greater than that in the US National Healthcare Safety Network hospitals, but 33% lower than that in the National Health Commission of China hospitals [9].

Certain bacteria are multidrug-resistant (MDR), indicating that they are resistant to several antibiotics. MDR bacteria can be difficult to treat because they may carry multiple genes that confer resistance to antibiotics; moreover, they can propagate these genes [10]. According to the World Health Organization (WHO), antibiotic-resistant microorganisms, including bacteria, are capable of resisting antimicrobial attacks, which contributes to the inadequate treatment of infections and results in the persistence and spread of pathogens. While the emergence of MDR bacteria is common, a significant increase in the number of patients with immunocompromised conditions—such as those who have undergone organ transplantation or have serious burns, end-of-life patients, and those at risk of contracting diseases—who are easy targets for hospital-acquired infectious diseases, has led to the further distribution of multidrug resistance. Studies by the WHO have reported high rates of resistance [11]. In the United States and Europe, patients develop infections caused by resistant bacteria every ten minutes. The spectrum of illnesses and trends of BSIs due to antimicrobial resistance vary among different world regions due to apparent epidemiological factors and regional variations [12].

The propagation of bacterial resistance is a major public health problem that may contribute to BSIs. In fact, in the last few years, the prevalence of BSIs resistant to frequently used antimicrobials has increased, and improper drug usage has also contributed to antibiotic resistance [13]. For example, the overuse of antimicrobials in ICUs was recorded in a hospital-based study in Saudi Arabia in 2010, and counter sales of local drugs in Saudi Arabia were high; thus, these were found to be important reasons for the development and spread of antimicrobial resistances [12]; therefore, population routine testing is required.

Antimicrobial treatment must be provided for a reasonable period of time to prevent BSI recurrence; however, prolonged antimicrobial therapy is associated with the development of antimicrobial resistance, which is rapidly increasing worldwide. Consequently, the WHO released the global action plan on antimicrobial resistance in 2015, with Saudi Arabia as a signatory. One objective of this plan was to optimize the use of antimicrobial agents [14]. Unfortunately, the occurrence of a series of pandemics over the last two decades has increased multidrug resistance in multiple bacterial pathogens. In the United States, over two million individuals are estimated to be infected with antibiotic-resistant bacteria annually, and at least 23,000 people succumb to these infections, as indicated by the Centers for Disease Control and Prevention [15]. The cost of treating resistant microbes is projected to reach USD 20 billion, with increased costs in direct healthcare expenses. A similar study in the UK forecasts 10 million annual deaths and up to USD 100 trillion in losses to the world economy by 2050. Globally, the effectiveness of antibiotic treatments will soon be reduced by 30% [16].

Extended-spectrum β-lactamases (ESBLs) are bacterial enzymes that hydrolyze clinically important antibiotics, and ESBL-producing bacterial infections are becoming increasingly difficult to treat. A progressively disturbing pattern worldwide is that of asymptomatic carriers of ESBL-producing bacteria. According to recent estimates, ESBL-producing bacteria colonize more than 50% of the population in some areas of Southeast Asia, and this number is even higher in other parts of the world; Therefore, millions of people are at risk of developing antibiotic-resistant infections [17]. The most common ESBL-producing gram-positive bacteria are *Staphylococcus aureus* and coagulase-negative *Staphylococcus* (CoNS), whereas the most common ESBL-producing gram-negative bacteria are *Escherichia coli*, *Klebsiella pneumoniae*, and *Pseudomonas aeruginosa* [8].

Infections caused by gram-negative bacteria are a significant health problem worldwide. *Enterobacteriaceae* resistance to cephalosporins and carbapenems indicates that third-generation antibiotics are needed to ensure efficient and prompt management of these bacteria. In general, the clinical outcomes of patients with BSIs due to the resistance of *E. coli* and *K. pneumoniae* to carbapenem are poor, with a mortality rate of 50%. In addition, a multidrug resistance rate of 69% was found in a recent analysis of *Acinetobacter baumannii* in Aseer province, Saudi Arabia; however, only 0.05% and 0.04% of imipenem (IMP)- and meropenem (MER)-resistant bacteria, respectively, were identified [18]. The most common pathogens causing BSIs in South Korea from 2016 to 2017 were *E. coli* and *S. aureus*, and the European Antimicrobial Resistance Surveillance Network published similar results from 2002 to 2009 [12].

Antimicrobial-resistant gram-negative bacteria, such as *E. coli*, *P. aeruginosa*, *Acinetobacter*, and *Klebsiella* spp., have spread to ICUs as causative agents of nosocomial infections over the past decade. They are a common cause of bloodstream, urinary, postoperative, skin, and soft tissue infections. The risk of acquiring nosocomial infections caused by antibiotic-resistant pathogens is higher in patients in ICUs than in those in other hospital areas, and MDR bacterial infections are the main cause of morbidity and mortality. Moreover, the rate of appearance of MDR bacterial patterns for gram-negative pathogens in patients admitted to ICUs is several times higher than that in patients treated in other hospital units; therefore, the spread of these pathogens is a global health concern.

Global surveillance studies have been conducted to identify bacterial pathogens in ICUs and determine their resistance profiles. In tertiary hospitals in Saudi Arabia, multiple local studies have followed the concentrations of resistant pathogens in these wards; However, this information is scarce in some areas such as the province of Bisha in southwestern Saudi Arabia. To develop protocols for the effective treatment of infectious agents, obtaining regional statistics on resistance rates is vital. Overall, gram-negative bacteria showed a high rate of resistance to cefuroxime (CXM; 75.8%), trimethoprim-sulfamethoxazole (SXT; 73.4%), cefotaxime (CTX; 72.9%), aztreonam (AZT; 64.6%), piperacillin-tazobactam (TZP; 62.1%), and ciprofloxacin (CIP; 61.5%). Specifically, *Acinetobacter* showed a high resistance (from 93.4% to 97.5%) to all antimicrobials, whereas *K. pneumoniae* showed a high resistance to SXT (71.8%), CTX (71.4%), and AZT (65.2%) [19].

This study aimed to elucidate the overall prevalence of MDR bacteria in the bloodstream of patients hospitalized in King Fahd Medical City, Riyadh, Saudi Arabia, between 1 January and 31 December 2020. In addition, we determined the general prevalence of bacteria in the bloodstream by age and sex and the antibiotic resistance rates of the identified MDR bacteria.

## 2. Materials and Methods

### 2.1. Collection of Blood Specimens

This study was conducted between January and December 2020 in Riyadh, Saudi Arabia. The sample size consisted of 1039 specimens from the same number of patients; samples were collected by members of the Department of Microbiology, King Fahd Medical City, Riyadh, Saudi Arabia. Blood specimen collection and preservation methods were established in accordance with international standards. Patient information was recorded using the electronic system of King Fahd Medical City. Clinicians collected data on age, ethnicity, tribe, residence, educational level, and medical history of the patients. Blood samples were collected after obtaining informed consent from all patients. The samples were divided into groups according to patient age (pediatric [0–12 years], adult [13–64 years], and elderly [>64 years]) and sex at birth.

### 2.2. Isolation and Identification of Bacteria

Bacterial pathogens were isolated and identified at the Department of Microbiology of King Fahd Medical City, Riyadh, Saudi Arabia. Blood samples were pretested to detect microbial growth using an automatic blood culture device BD BACTEC™ FX (Becton, Dickinson and Company, Franklin Lakes, NJ, USA) [20], and those with positive signals for microbial growth were used to isolate bacterial pathogens. The samples were then cultured on blood agar, chocolate agar, McConkey agar, and mannitol salt agar plates at 37 °C under aerobic conditions for 18 h. After incubation, the bacterial cultures were purified on blood agar plates until pure cultures were obtained [21]. Isolated bacteria were tested by Gram staining [22], and both gram-negative and gram-positive isolates were identified using the BD Phoenix system (BD Diagnostics, Baltimore, MD, USA), according to the manufacturer’s instructions. The system was designed for both rapid identification (ID) (45 wells with dried biochemical substrates and two fluorescent control wells) and antimicrobial susceptibility testing (up to 84 wells with dried antimicrobial panels) of bacterial isolates. This system uses modified traditional fluorogenic and chromogenic substrates as redox indicators to detect bacterial growth in the presence of antimicrobial agents [23]. Briefly, the ID broth was independently inoculated with pure culture bacterial colonies calibrated to a McFarland range of 0.5–0.6 using a CrystalSpec nephelometer (BD Diagnostics). A 25 mL aliquot of this suspension was withdrawn for antimicrobial susceptibility testing, and the remaining suspension was poured onto the ID side of the Phoenix panel. Antimicrobial susceptibility was determined according to the Clinical and Laboratory Standards Institute (CLSI) guidelines [24]. Valid identification of the isolates required a score greater than 90%; otherwise, no identification was recorded.

### 2.3. Antimicrobial Susceptibility of Clinical Isolates

As previously mentioned, all the clinical isolates were tested for antimicrobial susceptibility using the BD Phoenix™ system. Bacterial complete resistance (resistance), intermediate resistance (intermediate), and susceptibility were determined for the following antimicrobials: amoxicillin-clavulanate (AM-CLAV), ampicillin (AMP), amikacin (AN), aztreonam (AZT), ceftazidime (CAZ), cephalothin (CEF); cefalotin (CF), ciprofloxacin (CIP), chloramphenicol (CL), ceftolozane-tazobactam (CPE), cefotaxime (CTX), cefuroxime (CXM), ertapenem (ERT), imipenem (IMP), gentamicin (GM), levofloxacin (LEVO), meropenem (MER), penicillin (PEN), trimethoprim-sulfamethoxazole (SXT), piperacillin-tazobactam (TZP), clindamycin (CC), erythromycin (ERY), oxacillin (OX), and vancomycin (VA).

### 2.4. Data Analysis

The data were statistically analyzed using the Minitab Lab Manual. Values were considered statistically significant at the 95% confidence level and *p* < 0.05.

### 2.5. Ethical Consideration

This study was approved by the Research Committee of the King Fahad Medical City Medical Research Center, Riyadh, Saudi Arabia (IRB Log No. 21-002E).

## 3. Results

### 3.1. Isolation and Identification of Clinical Bacterial from Targeted Patients

In the present study, 1039 blood samples were cultured on different selective media to isolate clinical bacteria. In total, 1125 bacterial isolates were identified using the BD Phoenix™ system (Table 1). The bacterial isolates were classified in accordance with their gram characteristics into 629 (55.90% of the total) gram-positive bacteria, namely, *Bacillus* spp., *Enterococcus avium*, *Enterococcus casseliflavus*, *Enterococcus durans*, *Enterococcus faecalis*, *Enterococcus faecium*, *Enterococcus raffinosus*, methicillin-sensitive *Staphylococcus aureus* (*S. aureus*), methicillin-resistant *Staphylococcus aureus* (MRSA), *Staphylococcus capitis*, *Staphylococcus caprae*, *Staphylococcus cohnii*, *Staphylococcus epidermidis*, *Staphylococcus haemolyticus*, *Staphylococcus hominis*, *Staphylococcus lentus*, *Staphylococcus lugdunensis*, *Staphylococcus pettenkoferi*, *Staphylococcus saprophyticus*, *Staphylococcus schleiferi*, *Staphylococcus sciuri*, *Staphylococcus simulans*, *Staphylococcus* spp., CoNS, *Staphylococcus warneri*, *Streptococcus acidominimus*, *Streptococcus agalactiae*, *Streptococcus anginosus*, *Streptococcus constellatus*, *Streptococcus milleri* group bacteria, *Streptococcus mitis*, *Streptococcus oralis*, *Streptococcus parasanguinis*, *Streptococcus pneumoniae*, *Streptococcus pyogenes*, *Streptococcus sanguis*, *Streptococcus viridans*, group C *Streptococcus*, *Corynebacterium* group JK, *Gemella* spp., *Lactobacillus* spp., *Leuconostoc* spp., *Pediococcus pentosaceus*, and *Nocardia* spp. (Table 1); and 496 (44.08% of the total) gram-negative bacterial isolates, including *Achromobacter* spp., *Acinetobacter baumanni*, *Acinetobacter lwoffi*, *Aeromonas caviae*, *Aeromonas hydrophila*, *Aeromonas veronii*, *Alcaligenes denitrificans*, *Burkholderia cepacia*, *Brucella* spp., *Citrobacter amalonaticus*, *Citrobacter farmeri*, *Citrobacter freundii*, *Citrobacter koseri*, *Citrobacter* spp., *Campylobacter* spp., *Eikenella corrodens*, *Elizabethkingia meningoseptica*, *Enterobacter cloacae*, *Enterobacter gergoviae*, *Escherichia coli* (non-ESBL), *Escherichia coli* (ESBL), *Escherichia vulneris*, *Haemophilus influenzae*, *Klebsiella aerogenes*, *Klebsiella oxytoca*, *Klebsiella ozaenae*, *Klebsiella pneumoniae* (non-ESBL), *Klebsiella pneumoniae* (ESBL), *Kluyvera ascorbata*, *Morganella morganii*, *Pantoea agglomerans*, *Proteus mirabilis* (non-ESBL), *Proteus mirabilis* (ESBL), *Providencia rustigianii*, *Providencia stuartii*, *Providencia rettgeri*, *Pseudomonas aeruginosa*, *Pseudomonas fluorescens*, *Salmonella* group D (non-*typhi*), *Salmonella* spp. (non-*typhi*), *Serratia marcescens*, *Serratia plymuthica*, *Shigella flexneri*, *Sphingomonas paucimobilis*, and *Neisseria* spp. (Table 1). The number of gram-positive bacterial isolates was significantly higher than that of gram-negative isolates (*p* = 0.0001; 95% confidence interval [CI]; Table 1).

### 3.2. Distribution of Isolated Clinical Bacteria in Relation to Patient Age

**Figure 1:**
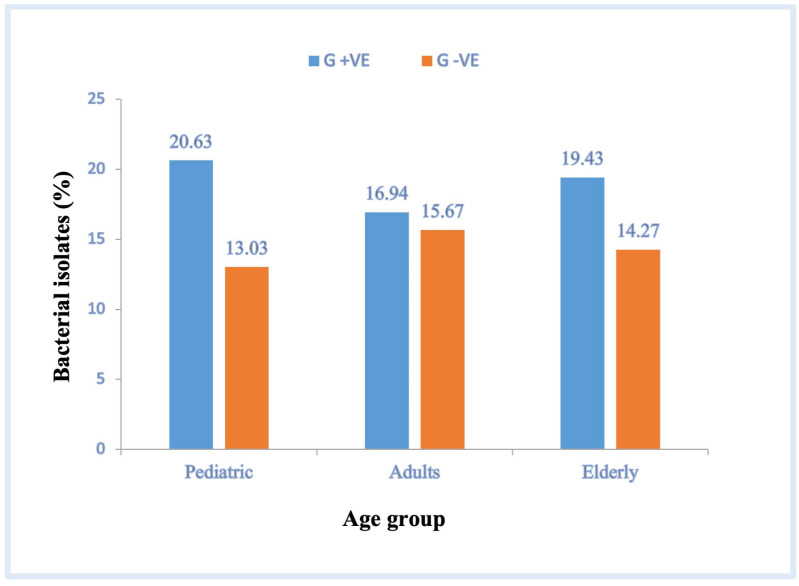
Percentage of isolated clinical gram-positive and gram-negative bacteria per patient age. G +ve, gram-positive bacteria; G −ve, gram-negative bacteria.

We analyzed the distribution of isolated clinical bacteria according to patient age (Table 2 and Table 3). Most gram-negative bacterial species were isolated from adult patients (15.67%); however, 13.03% and 14.27% of the gram-negative bacterial species were isolated from pediatric and elderly patients, respectively (Figure 1). The results in Table 2 show that the prevalence of bacterial pathogens isolated from male and female pediatric patients was 14.01% and 16.41% and from male and female adult patients was 17.50% and 19.12%, and that from male and female elderly patients was 15.75% and 17.17%, respectively, with no significant differences in the number of clinical bacterial isolates between males and females in either group (*p* = 0.257, 0.241, and 0.525, respectively; 95% CI). In general, the prevalence of gram-negative bacterial isolates was higher among adult patients than among other patient groups. The most prevalent gram-negative bacterial isolates in the three patient groups were *E. cloacae*, *E. coli* (non-ESBL), *E. coli* (ESBL), *K. pneumoniae* (non-ESBL), *K. pneumoniae* (ESBL), *S. marcescens*, and *A. baumannii* (Table 2).

Our results showed that gram-positive bacterial isolates were significantly more abundant in pediatric patients (~21%) than in elderly and adult patients (19.43% and 16.94%, respectively) (*p* = 0.037, 95% CI; Figure 1). The results also showed that the proportion of gram-positive bacterial isolates in male pediatric patients was slightly higher than that in adult and elderly male patients (19.49%, 15.05%, and 17.57%, respectively; Table 3). However, the prevalence of gram-positive bacterial isolates in pediatric and elderly female patients was higher than that in adult female patients (16.27%, 16.59%, and 15.01%, respectively). The results in Table 3 show that the prevalence of isolated bacterial pathogens was significantly higher in male pediatric patients than in female pediatric patients (19.49% and 16.27%, respectively; *p* = 0.037, 95% CI). In contrast, no significant differences were found between the prevalence rates of isolated gram-positive pathogens in male and female adults (15.05% and 15.01%, respectively; *p* = 0.895, 95% CI) and between those in male and female elderly patients (17.57% and 16.59%, respectively; *p* = 0.555; 95% CI). The most commonly isolated gram-positive bacteria from all three groups were *E. faecalis*, *S. aureus*, MRSA, *S. epidermidis*, *S. hominis*, and *S. viridans* (Table 3).

### 3.3. Isolated Clinical Bacteria Percentage Differences in Relation with Patient’s Sex at Birth

**Figure 2:**
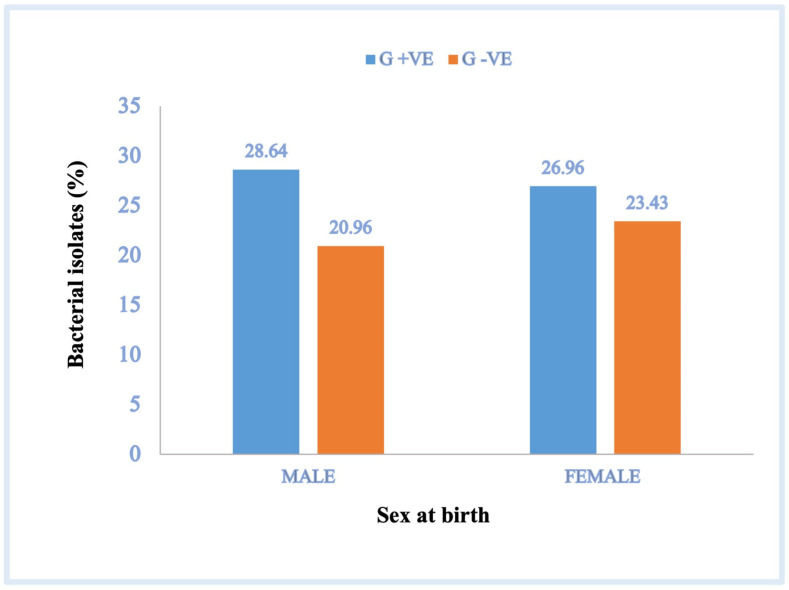
Percentage of clinical gram-positive and gram-negative bacterial isolates per patient sex. G +ve, gram-positive bacteria; G −ve, gram-negative bacteria.

This study also focused on the association between clinical bacterial pathogens and male and female-targeted patients (Table 4 and Table 5). Significantly more clinical bacterial isolates were obtained from female patients (50.39%) than from male patients (49.60%) (*p* = 0.037, 95% CI; Figure 2), and the prevalence of gram-negative isolates (20.96%) was significantly lower than that of gram-positive isolates (28.64%) in male patients (*p* = 0.001, 95% CI). A similar phenomenon was observed for the prevalence of gram-negative (23.43%) and gram-positive (26.96%) bacteria in female patients (*p* = 0.022, 95% CI; Figure 2). The order of prevalence of bacterial pathogens isolated from female patients was *S. epidermidis* (9.42%), *K. pneumoniae* (4.8%), *E. coli* (non-ESBL) (3.2%), *P. aeruginosa* (2.57%), *S. hominis* (2.40%), MRSA (2.13%), *E. coli* (ESBL) (1.95%), *S. aureus* (1.95%), *E. cloacae* (1.6%), *E. faecalis* (1.33%), *K. pneumoniae* (ESBL) (1.24%), *S. viridans* (1.24%), *S. capitis* (0.97%), *S. marcescens* (0.97%), *A. baumanni* (0.71%), and *E. faecium* (0.60%), whereas, in male patients, the order was *S. epidermidis* (11.02%), *K. pneumoniae* (non-ESBL) (6.75%), *S. hominis* (3.80%), *S. aureus* (2.93%), *E. coli* (non-ESBL) (2.93%), *S. capitis* (2.40%), *P. aeruginosa* (2.22%), MRSA (2.04%), *E. faecalis* (1.86%), *K. pneumoniae* (ESBL) (1.68%), *E. coli* (ESBL) (1.51%), *S. viridans* (1.42%), *E. cloacae* (1.42%), *A. baumannii* (1.33%), *E. faecium* (1.24%), and *S. marcescens* (0.80%), as shown in Table 3 and Table 4.

### 3.4. Susceptibility and Resistance of the Most Prevalent Clinical Bacterial Isolates to the Tested Antimicrobials

In this study, all gram-positive bacterial isolates were tested for CF, CTX, CEF, CAZ, CPE, AMP, AM-CLAV, OX, PEN, GM, CIP, ERY, CC, SXT, and VA resistance, whereas all gram-negative bacterial isolates were tested for CF, CXM, CTX, CEF, CAZ, CPE, AMP, AM-CLAV, PEN, GM, AN, CIP, LEVO, IMP, MER, ERT, AZT, CL, TZP, and SXT resistance.

**Figure 3:**
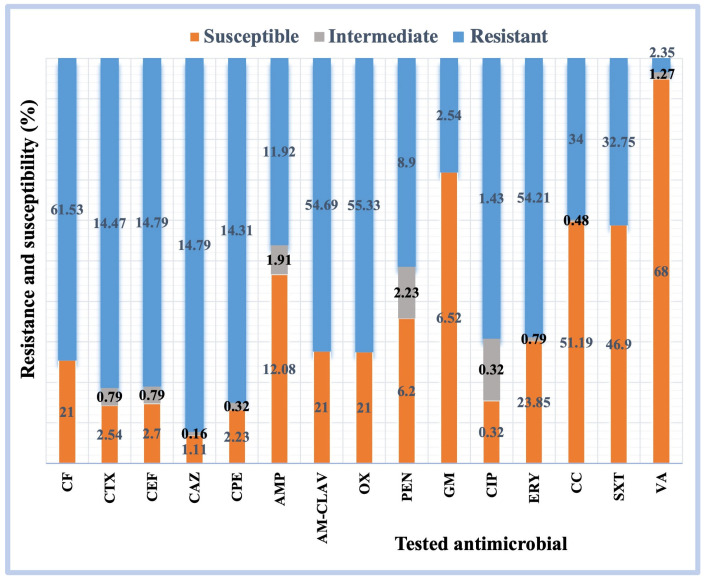
Susceptibility and resistance to a variety of antibiotics among isolated gram-positive bacteria. AM-CLAV, amoxicillin-clavulanate; AMP, ampicillin; CAZ, ceftazidime; CC, clindamycin; CEF, cephalothin; CF, cefalotin; CIP, ciprofloxacin; CPE, ceftolozane-tazobactam; CTX, cefotaxime; ERY, erythromycin; GM, gentamicin; OX, oxacillin; PEN, penicillin; SXT, trimethoprim-sulfamethoxazole; VA, vancomycin.

**Figure 4:**
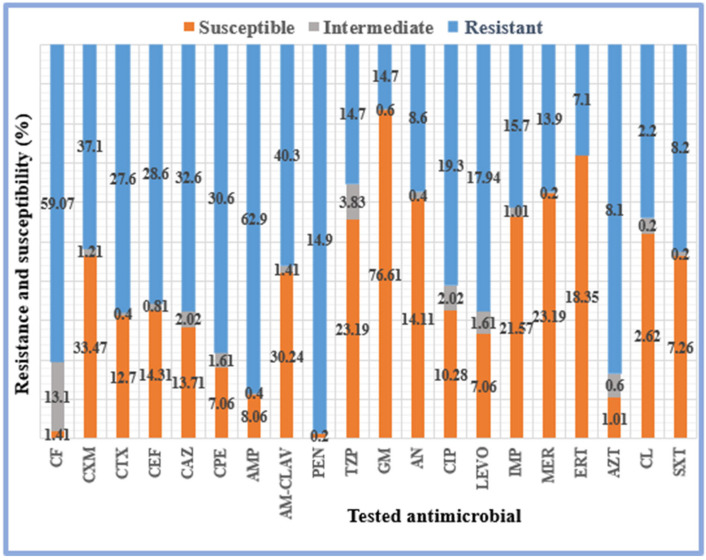
Susceptibility and resistance to a variety of antibiotics among isolated gram-negative bacteria. AM-CLAV, amoxicillin-clavulanate; AMP, ampicillin; AN, amikacin; AZT, aztreonam; CAZ, ceftazidime; CEF, cephalothin; CF, cefalotin; CIP, ciprofloxacin; CL, chloramphenicol; CPE, ceftolozane-tazobactam; CTX, cefotaxime; CXM, cefuroxime; ERT, ertapenem; IMP, imipenem; GM, gentamicin; LEVO, levofloxacin; MER, meropenem; PEN, penicillin; SXT, trimethoprim-sulfamethoxazole; TZP, piperacillin-tazobactam.

The resistance, intermediate, and susceptibility percentages of the gram-positive and gram-negative bacterial isolates to the targeted antimicrobials were determined (Figure 3 and Figure 4). High percentages of the gram-positive bacterial isolates were resistant to CF (61.5%), AM-CLAV (54.6%), OX (55.3%), and ERY (54.2%) (Figure 3). Moreover, high percentages of gram-negative bacteria were resistant to AMP (62.9%), CF (59.1%), and AM-CLAV (40.3%) (Figure 4). Notably, all the isolated bacteria showed high levels of resistance to a wide range of antimicrobials.

Our results (Table 6) showed that isolated MRSA and *E. faecalis* were highly resistant to antibiotics, especially to CF, CTX, CEF, CAZ, CPE, and AMP, with 100% resistance; MRSA was also 100% resistant to AM-CLAV, OX, and PEN, whereas *E. faecalis* only showed 100% resistance to CC. In contrast, MRSA was sensitive to VA (100%), CC (80.8%), ERY (72.3%), and SXT (70.2%), and *E. faecalis* was sensitive to AMP (97.2%), VA (77.7%), GM (66.6%), and CIP (2.77%). The isolated strains of *S. epidermidis*, *S. hominis*, *S. aureus*, *S. capitis*, *E. faecalis*, and *S. haemolyticus* were sensitive to VA, whereas *E. faecium* and *S. viridans* were only 57.1% and 3.3% sensitive to this antibiotic, respectively. Besides, *S. epidermidis* isolates were 87.4% resistant to each of CF, AM-CLAV, and OX, 84.1% to ERY, 57.2% to CC, 56.2% to SXT, 0.93% to GM, and 0.93% to CIP. *S. hominis* isolates were 80% resistant to ERY, 74.2% to OX, 72.8% to CF, 71.4% to AM-CLAV, 57.1% to SXT, 7.1% to CC, and 1.4% to CIP. The resistance percentages of *S. aureus* isolates to the tested antimicrobials were 32.7%, 9.1%, 5.5%, 1.8%, 1.8%, and 1.8% to ERY, CC, SXT, CF, AM-CLAV, and OX, respectively. Additionally, the resistance percentages of *S. capitis* isolates against the tested antimicrobials were 71.1% to ERY, 68.4% to CF, 68.4% to OX, 42.1% to CC, 26.3% to SXT, 6.15% to AM-CLAV, and 5.2% to CIP, whereas those of *S. haemolyticus* isolates were 77.7% to ERY, 74.1% to each CF, AM-CLAV, and OX, 33.3% to SXT, 29.6% to CC, and 3.7% to CIP. Our results also revealed that *E. faecium* isolates were 85.7% resistant to AMP, 23.8% to GM, and 4.7% to CIP. Among *S. viridans* isolates, 20% were resistant to AMP, 16.7% to CTX, 16.7% to CEF, 16.6% to PEN, and 13.3% to CPE.

The present study also determined that, among the isolated gram-negative bacterial strains, *E. coli* (ESBL) and *K. pneumoniae* (ESBL) were highly resistant, particularly to CF, CXM, CTX, CEF, CAZ, CPE, AM-CLAV, and PEN, with 100% resistance. However, while *E. coli* (ESBL) was 100% resistant to AMP, *K. pneumoniae* (ESBL) was only 3.3% resistant to this antibiotic. Moreover, *E. coli* (ESBL) and *K. pneumoniae* (ESBL) showed lower percentages of resistance to other antimicrobials: 2.5% and 6.0% to TZP, 25.6% and 30.3% to GM, 43.5% and 12.1% to CIP, 41% and 9.09% to LEVO, and 2.5% and 3.03% to SXT, respectively (Table 7). Our results also showed that the susceptibility of *E. coli* (ESBL) to IMP, MER, and ERT was 100%, and of 51.3%, 33.3%, 5.1%, and 5.1% to GM, AN, LEVO, and CIP, respectively. *K. pneumoniae* (ESBL) sensitivities to GM, AM-CLAV, CXM, AN, and CIP were 86.92%, 73.1%, 69.23%, 10%, and 3.85%, respectively (Table 7).

Our results also revealed that *K. pneumoniae* (non-ESBL) isolates were 96% resistant to AMP, 55% to CF, 26% to AM-CLAV, 24% to each TZP and CXM, 23% to each CTX, CEF, CAZ, and CPE, 22% to SXT, 21% to each CIP, LEVO, and IMP, 20% to MER, 19% to ERT, 18% to each AN and AZT, and 12% to GM, whereas *E. coli* (non-ESBL) isolates were 84% resistant to CF, 23% to AM-CLAV, 16% to CXM, 12% to CIP, 10% to CTX, 10% to CEF, 8.7% to CAZ, 7.2% to CPE, 17% to AMP, 8.7% to TZP, 8.7% to LEVO, 7.2% to GM, 4.3% to AZT, 2.9% to IMP, 2.9% to ERT, 2.9% to SXT, 1.4% to MER, and 1.4% to AN, as shown in Table 7.

*P. aeruginosa* and *E. cloacae* isolates (Table 7) also showed a wide range of resistance to the tested antimicrobials. *P. aeruginosa* isolates showed 28% resistance to each MER and IMP, 20% to CAZ, 19% to CPE, 11% to LEVO, 9.3% to CIP, 7.4% to TZP, 5.6% to GM, and 5.6% to AZT, whereas *E. cloacae* isolates showed 94% resistance to AMP, 91% to each CF and AM-CLAV, 77% to CXM, 24% to each CTX and CEF, 8.8% to each CAZ, CPE, CIP, and LEVO, 5.8% to each TZP and GM, and 2.9% to each IMP, MER, ERT, AZT, and SXT. The percentages of resistance observed for *A. baumanni* isolates against the tested antimicrobials were 74% to each CAZ, CPE, and TZP, 70% to each CIP, LEVO, IMP, and MER, 57% to GM, and 39% to AN, whereas, those of *S. marcescens* isolates to the tested antimicrobials were 100% to each AMP, AM-CLAV, and ERT, 95% to each CF and CXM, 10% to each CTX, CEF, IMP, and MER, 5% to AZT, and 0.5% to TZP (Table 7).

## 4. Discussion

This work focused on the isolation and detection of clinical bacterial strains from human blood specimens. Samples from 1039 patients from the Riyadh region of Saudi Arabia were used, and our results showed an abundance of 55.90% gram-positive bacteria and 44.10% gram-negative bacteria. These results are consistent with those of previous studies that found abundances of 71.8% and 28.2% for gram-positive and gram-negative bacterial strains isolated from 1618 blood specimens in China [25], of 47.3% and 47.3% for microbial isolates from Pakistani samples [26], and of 36.4% and 62.2% for microbial isolates from samples from the Aljouf region of Saudi Arabia [12], respectively.

In our study, the most frequently isolated gram-negative bacteria were *E. cloacae*, *E. coli* (non-ESBL), *E. coli* (ESBL), *K. pneumoniae* (non-ESBL), *K. pneumonia* (ESBL), *S. marcescens*, and *A. baumannii*, while the most frequently isolated gram-positive bacteria were *E. faecalis*, MRSA, *S. epidermidis*, *S. hominis*, and *S. viridans.* These findings are in agreement with those of Tian et al. [27], who isolated several clinical bacterial strains from Chinese patient blood samples, including *S. aureus*, *E. coli*, *K. pneumoniae*, and *S. typhi*. In addition, Ozbak [8] showed that the most commonly isolated bacteria in Saudi Arabia were *S. aureus* and CoNS, among the gram-positive, and *E. coli*, *K. pneumoniae*, and *P. aeruginosa*, among the gram-negative. Moreover, Akova [16] reported that *E. faecium*, *S. aureus*, *K. pneumoniae*, *A. baumannii*, *P. aeruginosa*, *E. coli*, and *Enterobacter* spp. are among the most frequently isolated bacteria from blood samples.

In pediatric, adult, and elderly patients, the most prevalent bacterial isolates were *E. cloacae*, *E. coli* (non-ESBL), *E. coli* (ESBL), *K. pneumoniae* (non-ESBL), *K. pneumoniae* (ESBL), *S. marcescens*, *A. baumannii*, *E. faecalis*, *S. aureus*, MRSA, *S. epidermidis*, *S. hominis*, and *S. viridans*. However, in adult patients, our results showed that the prevalence of gram-negative and gram-positive bacterial species was 15.67% and 16.94%, respectively. The prevalence of gram-negative bacterial isolates in male and female adult patients was 17.50% and 19.12%, respectively, and that of gram-positive bacterial isolates was 15.05% and 15.01%, respectively. Similarly, previous findings [28] showed that the percentages of gram-negative and gram-positive bacteria in adults were 51% and 42%, respectively, whereas the percentage of bacterial isolates from adults was 61% in male and 39% in female patients, with *E. coli*, *Klebsiella* spp., *Enterobacter* spp., *Serratia* spp., *Pseudomonas* spp., *Stenotrophomonas maltophilia*, *Acinetobacter* spp., *Salmonella* spp., CoNS, *S. aureus*, *Enterococcus* spp., *S. viridans*, *Corynebacterium jeikeium*, *S. agalactiae*, and *S. pneumoniae* as the most commonly isolated bacteria [28].

In this study, gram-negative and gram-positive bacterial isolates were found in 13.03% and 20.63% of the pediatric patients, respectively. In addition, the prevalence of gram-negative bacterial isolates in male and female pediatric patients was 14.01% and 16.41%, respectively, whereas that of gram-positive bacterial isolates in male and female pediatric patients was 19.49% and 16.27%, respectively. Our findings are consistent with previous findings in pediatrics [5], which reported that the percentages of gram-negative and gram-positive bacterial isolates were 23% and 48%, respectively. However, the numbers of bacterial isolates in male and female patients were 59% and 41%, respectively, and the most commonly observed gram-negative and gram-positive bacterial isolates were *K. pneumoniae*, *E. cloacae*, *E. coli*, *P. aeruginosa*, *S. maltophilia*, CoNS, *E. faecalis*, *E. faecium*, *S. viridans*, and *S. aureus* [5].

In elderly patients, the prevalence of gram-negative and gram-positive bacterial species was 14.27% and 19.43%, respectively; the prevalence of gram-negative bacterial isolates in elderly male and female patients was 15.75% and 17.17%, respectively; and that of gram-positive bacterial isolates was 17.57% and 16.59%, respectively. In a study by Gavazzi et al. [29], the percentages of gram-negative and gram-positive bacterial isolates in elderly patients were 50.2% and 44.6%, respectively, and the percentages of bacterial isolates in male and female elderly patients were 53% and 47%, respectively. The most commonly isolated bacteria were *Enterococcus* spp., *S. aureus*, *S. epidermidis*, *S. pneumoniae*, *E. coli*, *Klebsiella* spp., *Proteus* spp., and *Pseudomonas* spp. [29].

Considering our study objectives, we are keen to investigate the prevalence of anaerobic and difficult-to-grow bacteria by carrying out several relevant experiments in our future work. Our goal is to shed light on the complexity of these species and unearth insightful information in this specialist field. It is significant to notice that, even though these trials are scheduled for later investigation, we have already proactively included these theoretical considerations into our planned research. Our goal in adding these speculative possibilities is to create the groundwork for the upcoming studies that will be covered in a later work.

Importantly, infectious gram-positive and gram-negative multidrug-resistant bacteria are becoming more common in Riyadh, Saudi Arabia. It is crucial to keep in mind, however, the bacterial isolates obtained from clinical samples may not always be indicative of true infections, as they could also represent contamination or colonization. To solve this problem, we underline the importance of exercising caution when interpreting bacterial isolates from clinical samples. In our research, we now recognize the significance of distinguishing between pathogenic isolates and actual infections to prevent misunderstandings. The protocol in the hospital laboratory involves requesting a second blood culture bottle from the same patient if the initial blood sample yields a positive result. This precautionary measure is taken to ensure that any positive outcome is not a result of contamination. Additionally, the obtained result is cross-referenced with the patient’s medical condition for comparison.

In this study, antibiogram analyses of the gram-positive bacterial isolates showed that MRSA and *E. faecalis* strains were highly resistant, especially to CF, CTX, CEF, CAZ, CPE, AMP, AM-CLAV, OX, PEN, and CC, with 100% resistance. These results are similar to those of related studies on the antimicrobial resistance of MRSA strains to PEN and OX at 100%, AMP at 92.5%, and AM-CLAV at 81.13% [30], and on the resistance of *E. faecalis* strains to ERY, AMP, and PEN at 53.4%, 11.4%, and 9.1%, respectively [31].

While the isolates of *E. faecium* and *S. viridans* were resistant to VA (57.1% and 3.3%, respectively), *E. faecium* isolates showed 85.7% resistance to AMP and 23.8% resistance to GM, and *S. epidermidis* isolates showed 87.4% resistance to each CF, AM-CLAV, and OX, 84.1% to ERY, 57.2% to CC, and 56.2% to SXT. This is consistent with the results from a recent study conducted in China, which found resistance to VA in *S. epidermidis* and *E. faecium* strains at frequencies of 0.13% and 4.1%, respectively, and resistance to ERY and CC at 45.2% each, GM at 10.2%, CIP at 56.1%, AMP at 91.5%, PEN at 92.5%, GM at 70.4%, and CIP at 90.1% in *E. faecium* isolates. *S. viridans* isolates were 70.4% resistant to ERY, whereas no resistance to AMP or PEN was detected among them [32].

Resistance to a wide variety of antimicrobials was also identified in *S. hominis* isolates at rates of 80%, 74.2%, 72.8%, 71.4%, and 57.1% to ERY, OX, CF, AM-CLAV, and SXT, respectively. Lourtet-Hascoët et al., reported the resistance of *S. hominis* isolates to a wide variety of antimicrobials, including 91.2% resistance to ERY, 68.3% to CC, and 52.6% to CIP [32].

Here, the percentage of resistance of *S. aureus* isolates to the tested antimicrobials was 32.7% for ERY, 9.1% for CC, 5.5% for SXT, and 1.8% each for CF, AM-CLAV, and OX. Previous studies have found that 34.3% of *S. aureus* isolates were resistant to ERY, 17.9% to CC, 9.4% to SXT, and 1.49% to AM-CLAV, with no resistance to OX [30].

*S. capitis* isolates were 71.1% resistant to ERY, 68.4% to each CF and OX, 42.1% to CC, and 26.3% to SXT. This study found that these isolates were more resistant to the tested antimicrobials than strains from previous studies, as they had found that resistance to the tested antimicrobials in *S. capitis* was 12.5% to ERY, 4.2% to each CC and SXT, 79.1% to PEN, and 4.2% to GM [32].

In *S. haemolyticus* isolates, resistance was 77.7% for ERY and 74.1% for CF, AM-CLAV, and OX, while a previous study reported that resistance to these antimicrobials was 95.1% for ERY, 52.5% for CC, and 84.4% for CIP [32]. In addition, the resistance rates of *S. viridans* isolates to the tested antimicrobials were 20% for AMP, 16.7% for CTX, 16.7% for CEF, and 16.6% for PEN, while previous studies reported a resistance of 70.4% to ERY and no resistance to AMP or PEN [32].

Antibiogram analyses of gram-negative bacterial isolates showed that *E. coli* (ESBL) and *K. pneumoniae* (ESBL) were fully resistant to CF, CXM, CTX, CEF, CAZ, CPE, AM-CLAV, and PEN. However, while *E. coli* (ESBL) was fully resistant to AMP, *K. pneumoniae* (ESBL) was only 3.3% resistant to this antibiotic. Previous studies showed that *E. coli* (ESBL) and *K. pneumoniae* (ESBL) were extremely immune to AMP, and showed 97.8% and 97.3% resistance to CEF, respectively. Their tolerances to other widely used antimicrobials were 42.7% and 65% to CAZ, 64.7% and 78% to CPE, 14.4% and 40.2% to AM-CLAV, 4.5% and 29.1% to TZP, and 78.9% and 47.7% to LEVO, respectively [33].

A total of 96% of *K. pneumoniae* (non-ESBL) isolates were resistant to AMP, 55% to CF, 26% to AM-CLAV, 24% to each TZP and CXM, 23% to each CTX, CEF, and CPE, 22% to SXT, 21% to each CIP, LEVO, and IMP, 20% to MER, 19% to ERT, 18% to each AN and AZT, and 12% to GM. Previously, the resistance of *K. pneumoniae* (non-ESBL) isolates was reported to be 100% to AMP, 87% to AM-CLAV, 81% to TZP, 78% to SXT, 75% to each CTX, CEF, CAZ, and CPE, 65% to CIP, 59% to each ERT and AZT, 56% to each LEVO and MER, 50% to IMP, 43% to AN, and 40% to GM [34].

Our findings indicated that 84% of *E. coli* (non-ESBL) isolates were resistant to CF, 23% to AM-CLAV, 16% to CXM, 12% to CIP, and 10% to CTX and CEF. Our findings are consistent with those of a previous study that found that *E. coli* isolates were 100% resistant to AMP, 94% to each CTX, CEF, CAZ, SXT, and CPE, 91% resistant to AM-CLAV, 79% to each LEVO and CIP, 76% to each TZP and AZT, 56% to GM, 44% to IMP, 47% to each ERT and MER, and 29.5% to AN [34].

Furthermore, our findings revealed that 28% of *P. aeruginosa* isolates were immune to MER, 28% to IMP, 20% to CAZ, and 19% to CPE. However, in previous studies, the resistance percentage of *P. aeruginosa* isolates to the tested antimicrobials was 0% for MER, IMP, and CIP; 33% for each CAZ and LEVO; and 50% for CPE [34].

In addition, 94% of *E. cloacae* isolates were immune to AMP, 91% to each CF and AM-CLAV, 77% to CXM, and 24% to each CTX and CEF. These findings are consistent with recent findings indicating that 100% of *E. cloacae* strains are resistant to AMP, AM-CLAV, AZT, and SXT, 67% to each CTX, CEF, and CAZ, 55% to each GM, CIP, and TZP, and 22% to CPE [34].

The current study also revealed that 74% of *A. baumannii* isolates were resistant to each CAZ, CPE, and TZP, 70% to each CIP, LEVO, IMP, and MER, 57% to GM, and 39% to AN. These findings are consistent with previous findings, which indicated that 100% of *A. baumannii* isolates were resistant to CPE, 83.4% to each CAZ, CIP, IMP, 75% to each TZP and MER, and 41.6% to LEVO [34].

It was also observed that *S. marcescens* isolates were 100% resistant to AMP, AM-CLAV, and ERT and 95% resistant to CF and CXM. These findings are consistent with those of a previous study, which reported that 87.3% of *S. marcescens* isolates were resistant to AMP, 92.4% to AM-CLAV, 11.8% to CEF, 1.6% to MER, and 2.4% to TZP [33].

## 5. Conclusions

In the Riyadh region of Saudi Arabia, the increasing number of bacteremias caused by MDR gram-positive and gram-negative bacteria has arisen as a significant health concern. Clinical settings now view this increasing rate as a problem requiring major efforts to prevent it from escalating and spreading. We have shown that changes in both bacterial abundance and resistance occur in patients exposed to illnesses caused by these organisms. The changes observed in the abundance of gram-positive and gram-negative strains indicate that the elderly population, together with public health and healthcare institutions, face enormous obstacles.

## Figures and Tables

**Table 1:** Names, numbers, and total percentage of clinical gram-positive and gram-negative bacteria isolated from patients with BSIs.

G +ve, total n_i_ = 629 (55.90%)	*Bacillus* sp. (1)	*Staphylococcus epidermidis* (230)	*Streptococcus acidominimus* (3)	*Streptococcus* group C (3)
	*Enterococcus avium* (1)	*Staphylococcus haemolyticus* (27)	*Streptococcus agalactiae* (5)	*Corynebacterium* group JK (4)
	*Enterococcus casseliflavus* (3)	*Staphylococcus hominis* (70)	*Streptococcus anginosus* (2)	*Gemella* spp. (2)
	*Enterococcus durans* (1)	*Staphylococcus lentus* (4)	*Streptococcus constellatus* (1)	*Lactobacillus* sp. (1)
	*Enterococcus faecalis* (36)	*Staphylococcus lugdunensis* (3)	*Streptococcus milleri* (1)	*Leuconostoc* sp. (1)
	*Enterococcus faecium* (21)	*Staphylococcus pettenkoferi* (3)	*Streptococcus mitis* (2)	*Pediococcus pentosaceus* (1)
	*Enterococcus raffinosus* (2)	*Staphylococcus saprophyticus* (2)	*Streptococcus oralis* (1)	*Nocardia* sp. (1)
	*Staphylococcus aureus* (55)	*Staphylococcus schleiferi* (2)	*Streptococcus parasanguinis* (2)	
	MRSA (47)	*Staphylococcus sciuri* (1)	*Streptococcus pneumoniae* (7)	
	*Staphylococcus capitis* (38)	*Staphylococcus simulans* (1)	*Streptococcus pyogenes* (2)	
	*Staphylococcus caprae* (3)	coagulase-negative *Staphylococcus* spp. (6)	*Streptococcus sanguis* (1)	
	*Staphylococcus cohnii* (1)	*Staphylococcus warneri* (1)	*Streptococcus viridans* (30)	
G −ve, total n_i_ = 496 (44.10%)	*Achromobacter* spp. (2)	*Citrobacter koseri* (1)	*Klebsiella oxytoca* (8)	*Pseudomonas aeruginosa* (54)
	*Acinetobacter baumanni* (23)	*Citrobacter* sp. (1)	*Klebsiella ozaenae* (4)	*Pseudomonas fluorescens* (1)
	*Acinetobacter lwoffi* (4)	*Campylobacter* spp. (2)	*Klebsiella pneumoniae* (non-ESBL) (130)	*Salmonella* group D (non-*typhi*) (1)
	*Aeromonas caviae* (3)	*Eikenella corrodens* (1)	*Klebsiella pneumoniae* (ESBL) (33)	*Salmonella* spp. (non-*typhi*) (10)
	*Aeromonas hydrophila* (1)	*Elizabethkingia meningoseptica* (2)	*Kluyvera ascorbata* (2)	*Serratia marcescens* (20)
	*Aeromonas veronii* (1)	*Enterobacter cloacae* (34)	*Morganella morganii* (1)	*Serratia plymuthica* (1)
	*Alcaligenes denitrificans* (1)	*Enterobacter gergoviae* (2)	*Pantoea agglomerans* (2)	*Shigella flexneri* (1)
	*Burkholderia cepacia* (1)	*Escherichia coli* (non-ESBL) (69)	*Proteus mirabilis* (non-ESBL) (8)	*Sphingomonas paucimobilis* (1)
	*Brucella* spp. (8)	*Escherichia coli* (ESBL) (39)	*Proteus mirabilis* (ESBL) (1)	*Neisseria* sp. (1)
	*Citrobacter amalonaticus* (1)	*Escherichia vulneris* (1)	*Providencia rustigianii* (2)	
	*Citrobacter farmeri* (1)	*Haemophilus influenzae* (3)	*Providencia stuartii* (3)	
	*Citrobacter freundii* (2)	*Klebsiella aerogenes* (8)	*Providencia rettgeri* (1)	

G +ve, gram-positive bacteria; G −ve, gram-negative bacteria; total n_i_, total number of isolates in each gram group; %, percentage of the total number of bacterial isolates. Numbers in parentheses indicate the total number of isolates from each species/group.

**Table 2:** Prevalence of clinical gram-negative bacterial isolates in patients of different age groups.

Gram-Negative Bacterial Isolate	Pediatric (from 0 to 12) n_p_ = 282		Adult (from 13 to 64) n_p_ = 508		Elderly (65 or More) n_p_ = 249	
	Male n_p_ = 166	Female n_p_ = 116	Male n_p_ = 296	Female n_p_ = 212	Male n_p_ = 132	Female n_p_ = 117
*Achromobacter* spp.	0.00%	1 (008.%)	0.00%	0.00%	1 (008.%)	0.00%
*Acinetobacter baumanni*	3 (0.26%)	1 (008.%)	10 (0.88%)	6 (0.53%)	2 (0.17%)	1 (008.%)
*Acinetobacter lwoffi*	2 (0.17%)	2 (0.17%)	0.00%	0.00%	0.00%	0.00%
*Aeromonas caviae*	0.00%	0.00%	1 (008.%)	0.00%	2 (0.17%)	0.00%
*Aeromonas hydrophila*	0.00%	0.00%	0.00%	1 (008.%)	0.00%	0.00%
*Aeromonas veronii*	0.00%	0.00%	0.00%	1 (008.%)	0.00%	0.00%
*Alcaligenes denitrificans*	0.00%	0.00%	0.00%	1 (008.%)	0.00%	0.00%
*Burkholderia cepacia*	0.00%	0.00%	0.00%	1 (008.%)	0.00%	0.00%
*Brucella* spp.	2 (0.17%)	1 (008.%)	5 (0.44%)	0.00%	0.00%	0.00%
*Citrobacter amalonaticus*	1 (008.%)	0.00%	0.00%	0.00%	0.00%	0.00%
*Citrobacter farmeri*	0.00%	0.00%	0.00%	0.00%	1 (008.%)	0.00%
*Citrobacter freundii*	0.00%	0.00%	0.00%	2 (0.17%)	0.00%	0.00%
*Citrobacter koseri*	0.00%	0.00%	0.00%	0.00%	1 (008.%)	0.00%
*Citrobacter* sp.	0.00%	0.00%	0.00%	1 (008.%)	0.00%	0.00%
*Campylobacter* spp.	2 (0.17%)	0.00%	0.00%	0.00%	0.00%	0.00%
*Eikenella corrodens*	0.00%	0.00%	1 (008.%)	0.00%	0.00%	0.00%
*Elizabethkingia meningoseptica*	1 (008.%)	0.00%	0.00%	0.00%	1 (008.%)	0.00%
*Enterobacter cloacae*	5 (0.44%)	6 (0.53%)	7 (0.62%)	10 (0.88%)	4 (0.35%)	2 (0.17%)
*Enterobacter gergoviae*	0.00%	1 (0.86%)	0.00%	0.00%	1 (0.76%)	0.00%
*Escherichia coli* (non-ESBL)	5 (0.44%)	3 (0.26%)	20 (1.77%)	20 (1.77%)	8 (0.71%)	13 (1.15%)
*Escherichia coli* (ESBL)	3 (0.26%)	2 (0.17%)	13 (1.15%)	11 (0.97%)	1 (008.%)	9 (0.80%)
*Escherichia vulneris*	0.00%	0.00%	1 (008.%)	0.00%	0.00%	0.00%
*Haemophilus influenzae*	1 (008.%)	2 (0.17%)	0.00%	0.00%	0.00%	0.00%
*Klebsiella aerogenes*	2 (0.17%)	0.00%	2 (0.17%)	0.00%	3 (0.26%)	1 (008.%)
*Klebsiella oxytoca*	2 (0.17%)	0.00%	3 (0.26%)	2 (0.17%)	1 (008.%)	0.00%
*Klebsiella ozaenae*	1 (008.%)	0.00%	3 (0.26%)	0.00%	0.00%	0.00%
*Klebsiella pneumoniae* (non-ESBL)	19 (1.68%)	12 (1.06%)	38 (3.37%)	30 (2.66%)	19 (1.68%)	12 (1.06%)
*Klebsiella pneumoniae* (ESBL)	9 (0.80%)	5 (0.44%)	7 (0.62%)	7 (0.62%)	3 (0.26%)	2 (0.17%)
*Kluyvera ascorbata*	1 (008.%)	0.00%	0.00%	1 (008.%)	0.00%	0.00%
*Morganella morganii*	0.00%	0.00%	0.00%	0.00%	0.00%	1 (008.%)
*Pantoea agglomerans*	1 (008.%)	0.00%	0.00%	1 (008.%)	0.00%	0.00%
*Proteus mirabilis* (non-ESBL)	0.00%	0.00%	4 (0.35%)	1 (008.%)	1 (008.%)	2 (0.17%)
*Proteus mirabilis* (ESBL)	0.00%	0.00%	0.00%	0.00%	1 (008.%)	0.00%
*Providencia rustigianii*	0.00%	0.00%	1 (008.%)	1 (008.%)	0.00%	0.00%
*Providencia stuartii*	0.00%	0.00%	2 (0.17%)	0.00%	0.00%	1 (008.%)
*Providencia rettgeri*	0.00%	0.00%	0.00%	0.00%	1 (008.%)	0.00%
*Pseudomonas aeruginosa*	3 (0.26%)	9 (0.80%)	17 (1.51%)	10 (0.88%)	5 (0.44%)	10 (0.88%)
*Pseudomonas fluorescens*	0.00%	0.00%	1 (008.%)	0.00%	0.00%	0.00%
*Salmonella* group D (non-*typhi*)	0.00%	0.00%	0.00%	0.00%	1 (008.%)	0.00%
*Salmonella* spp. (non-*typhi*)	1 (008.%)	4 (0.35%)	2 (0.17%)	3 (0.26%)	0.00%	0.00%
*Serratia marcescens*	1 (008.%)	4 (0.35%)	6 (0.53%)	5 (0.44%)	2 (0.17%)	2 (0.17%)
*Serratia plymuthica*	0.00%	0.00%	0.00%	0.00%	0.00%	1 (008.%)
*Shigella flexneri*	0.00%	0.00%	1 (008.%)	0.00%	0.00%	0.00%
*Sphingomonas paucimobilis*	0.00%	1 (008.%)	0.00%	0.00%	0.00%	0.00%
*Neisseria* sp.	1 (008.%)	0.00%	0.00%	0.00%	0.00%	0.00%
Total % of bacterial isolates	66 (14.01%)	54 (16.41%)	145 (17.50%)	115 (19.12%)	59 (15.75%)	57 (17.17%)

n_p_, number of patients; n (%), number of isolates (percentage of isolates per group).

**Table 3:** Prevalence of clinical gram-positive bacterial isolates in patients of different age groups.

Gram-Positive Bacterial Isolate	Pediatric (from 0 to 12) n_p_ = 282		Adult (from 13 to 64) n_p_ = 508		Elderly (65 or More) n_p_ = 249	
	Male n_p_ = 166	Female n_p_ = 116	Male n_p_ = 296	Female n_p_ = 212	Male n_p_ = 132	Female n_p_ = 117
*Bacillus* sp.	1 (0.08%)	0.00%	0.00%	0.00%	0.00%	0.00%
*Enterococcus avium*	0.00%	0.00%	1 (0.08%)	0.00%	0.00%	0.00%
*Enterococcus casseliflavus*	1 (0.08%)	0.00%	0.00%	0.00%	2 (0.17%)	0.00%
*Enterococcus durans*	1 (0.08%)	0.00%	0.00%	0.00%	0.00%	0.00%
*Enterococcus faecalis*	4 (0.35%)	2 (0.17%)	10 (3.42%)	7 (0.62%)	7 (0.62%)	6 (0.53%)
*Enterococcus faecium*	4 (0.35%)	1 (0.08%)	3 (0.26%)	3 (0.26%)	7 (0.62%)	3 (0.26%)
*Enterococcus raffinosus*	0.00%	0.00%	0.00%	2 (0.94%)	0.00%	0.00%
*Staphylococcus aureus*	8 (0.71%)	2 (0.17%)	19 (6.51%)	12 (1.06%)	6 (0.53%)	8 (0.71%)
MRSA	8 (0.71%)	7 (0.62%)	10 (0.88%)	13 (1.15%)	5 (0.44%)	4 (0.35%)
*Staphylococcus capitis*	5 (0.44%)	1 (0.86%)	13 (1.15%)	2 (0.17%)	9 (0.80%)	8 (0.71%)
*Staphylococcus caprae*	1 (0.08%)	0.00%	1 (0.08%)	1 (0.08%)	0.00%	0.00%
*Staphylococcus cohnii*	0.00%	0.00%	0.00%	0.00%	1 (0.08%)	0.00%
*Staphylococcus epidermidis*	47 (4.17%)	38 (3.37%)	52 (4.6%)	38 (3.37%)	30 (2.66%)	25 (2.22%)
*Staphylococcus haemolyticus*	0.00%	2 (0.17%)	13 (1.15%)	2 (0.17%)	8 (0.71%)	2 (0.17%)
*Staphylococcus hominis*	18 (1.60%)	6 (0.53%)	17 (1.51%)	10 (0.88%)	8 (0.71%)	11 (0.97%)
*Staphylococcus lentus*	1 (0.08%)	0.00%	1 (0.08%)	1 (0.08%)	1 (0.08%)	0.00%
*Staphylococcus lugdunensis*	0.00%	1 (0.08%)	1 (0.08%)	1 (0.08%)	0.00%	0.00%
*Staphylococcus pettenkoferi*	1 (0.08%)	0.00%	1 (0.08%)	0.00%	0.00%	1 (0.08%)
*Staphylococcus saprophyticus*	1 (0.08%)	0.00%	1 (0.08%)	0.00%	0.00%	0.00%
*Staphylococcus schleiferi*	0.00%	0.00%	2 (0.17%)	0.00%	0.00%	0.00%
*Staphylococcus sciuri*	1 (0.08%)	0.00%	0.00%	0.00%	0.00%	0.00%
*Staphylococcus simulans*	0.00%	0.00%	1 (0.08%)	0.00%	0.00%	0.00%
coagulase-negative *Staphylococcus* spp.	0.00%	0.00%	3 (0.26%)	1 (0.08%)	1 (0.08%)	1 (0.08%)
*Staphylococcus warneri*	1 (0.08%)	0.00%	0.00%	0.00%	0.00%	0.00%
*Streptococcus acidominimus*	1 (0.08%)	0.00%	0.00%	2 (0.17%)	0.00%	0.00%
*Streptococcus agalactiae*	1 (0.08%)	1 (0.08%)	1 (0.08%)	2 (0.17%)	0.00%	0.00%
*Streptococcus anginosus*	0.00%	0.00%	2 (0.17%)	0.00%	0.00%	0.00%
*Streptococcus constellatus*	0.00%	1 (0.08%)	0.00%	0.00%	0.00%	0.00%
*Streptococcus milleri*	0.00%	0.00%	0.00%	0.00%	1 (0.08%)	0.00%
*Streptococcus mitis*	0.00%	0.00%	1 (0.08%)	1 (0.08%)	0.00%	0.00%
*Streptococcus oralis*	1 (0.08%)	0.00%	0.00%	0.00%	0.00%	0.00%
*Streptococcus parasanguinis*	2 (0.17%)	0.00%	0.00%	0.00%	0.00%	0.00%
*Streptococcus pneumoniae*	2 (0.17%)	0.00%	0.00%	3 (0.26%)	1 (0.08%)	1 (0.08%)
*Streptococcus pyogenes*	1 (0.08%)	0.00%	0.00%	1 (0.08%)	0.00%	0.00%
*Streptococcus sanguis*	0.00%	0.00%	0.00%	1 (0.08%)	0.00%	0.00%
*Streptococcus viridans*	8 (0.71%)	3 (0.26%)	6 (0.53%)	10 (0.88%)	2 (0.17%)	1 (0.08%)
*Streptococcus* group C	0.00%	0.00%	2 (0.17%)	1 (0.08%)	0.00%	0.00%
*Corynebacterium* group JK	1 (0.08%)	0.00%	1 (0.08%)	2 (0.17%)	0.00%	0.00%
*Gemella* spp.	0.00%	0.00%	0.00%	1 (0.08%)	1 (0.08%)	0.00%
*Lactobacillus* sp.	0.00%	1 (0.08%)	0.00%	0.00%	0.00%	0.00%
*Leuconostoc* sp.	0.00%	0.00%	0.00%	0.00%	0.00%	1 (0.08%)
*Pediococcus pentosaceus*	1 (0.08%)	0.00%	0.00%	0.00%	0.00%	0.00%
*Nocardia* sp.	0.00%	0.00%	1 (0.08%)	0.00%	0.00%	0.00%
Total % of bacterial isolates	120 (19.49%)	70 (16.27%)	163 (15.05%)	118 (15.01%)	86 (17.57%)	72 (16.59%)

n_p_, number of patients; n (%), number of isolates (percentage of isolates per group).

**Table 4:** Difference in the abundance of gram-negative bacteria between male and female patients.

Gram-Negative Bacterial Isolate	Male Patients n_p_ = 594	Female Patients n_p_ = 445
*Achromobacter* spp.	1 (0.08%)	1 (0.08%)
*Acinetobacter baumanni*	15 (1.33%)	8 (0.71%)
*Acinetobacter lwoffi*	2 (0.17%)	2 (0.17%)
*Aeromonas caviae*	3 (0.26%)	0
*Aeromonas hydrophila*	0	1 (0.08%)
*Aeromonas veronii*	0	1 (0.08%)
*Alcaligenes denitrificans*	0	1 (0.08%)
*Burkholderia cepacia*	0	1 (0.08%)
*Brucella* spp.	7 (0.70%)	1 (0.08%)
*Citrobacter amalonaticus*	1 (0.08%)	0
*Citrobacter farmeri*	1 (0.08%)	0
*Citrobacter freundii*	0	2 (0.17%)
*Citrobacter koseri*	1 (0.08%)	0
*Citrobacter* sp.	0	1 (0.22%)
*Campylobacter* spp.	2 (0.17%)	0
*Eikenella corrodens*	1 (0.08%)	0
*Elizabethkingia meningoseptica*	2 (0.17%)	0
*Enterobacter cloacae*	16 (1.42%)	18 (1.6%)
*Enterobacter gergoviae*	1 (0.08%)	1 (0.08%)
*Escherichia coli* (non-ESBL)	33 (2.93%)	36 (3.2%)
*Escherichia coli* (ESBL)	17 (1.51%)	22 (1.95%)
*Escherichia vulneris*	1 (0.08%)	0
*Haemophilus influenzae*	1 (0.08%)	2 (0.17%)
*Klebsiella aerogenes*	7 (0.62%)	1 (0.08%)
*Klebsiella oxytoca*	6 (0.53%)	2 (0.17%)
*Klebsiella ozaenae*	4 (0.35%)	0
*Klebsiella pneumoniae* (non-ESBL)	76 (6.75%)	54 (4.8%)
*Klebsiella pneumoniae* (ESBL)	19 (1.68%)	14 (1.24%)
*Kluyvera ascorbata*	1 (0.08%)	1 (0.08%)
*Morganella morganii*	0	1 (0.08%)
*Pantoea agglomerans*	1 (0.08%)	1 (0.08%)
*Proteus mirabilis* (non-ESBL)	5 (0.44%)	3 (0.26%)
*Proteus mirabilis* (ESBL)	1 (0.08%)	0
*Providencia rustigianii*	1 (0.08%)	1 (0.08%)
*Providencia stuartii*	2 (0.17%)	1 (0.08%)
*Providencia rettgeri*	1 (0.08%)	0
*Pseudomonas aeruginosa*	25 (2.22%)	29 (2.57%)
*Pseudomonas fluorescens*	1 (0.08%)	0
*Salmonella* group D (non-*typhi*)	1 (0.08%)	0
*Salmonella* spp. (non-*typhi*)	3 (0.26%)	7 (0.62%)
*Serratia marcescens*	9 (0.80%)	11 (0.97%)
*Serratia plymuthica*	0	1 (0.08%)
*Shigella flexneri*	1 (0.08%)	0
*Sphingomonas paucimobilis*	0	1 (0.08%)
*Neisseria* sp.	1 (0.08%)	0
Total % of gram-negative bacteria	270 (20.96%)	226 (23.43%)

n_p_, number of patients; n (%), number of isolates (percentage of isolates in patients).

**Table 5:** Difference in the abundance of gram-positive bacteria between male and female patients.

Gram-Positive Bacterial Isolate	Male Patients n_p_ = 594	Female Patients n_p_ = 445
*Bacillus* sp.	1 (0.08%)	0
*Enterococcus avium*	1 (0.08%)	0
*Enterococcus casseliflavus*	3 (0.26%)	0
*Enterococcus durans*	1 (0.08%)	0
*Enterococcus faecalis*	21 (1.86%)	15 (1.33%)
*Enterococcus faecium*	14 (1.24%)	7 (0.6%)
*Enterococcus raffinosus*	0	2 (0.45%)
*Staphylococcus aureus*	33 (2.93%)	22 (1.95%)
MRSA	23 (2.04%)	24 (2.13%)
*Staphylococcus capitis*	27 (2.40%)	11 (0.97%)
*Staphylococcus caprae*	2 (0.17%)	1 (0.08%)
*Staphylococcus cohnii*	1 (0.08%)	0
*Staphylococcus epidermidis*	124 (11.02%)	106 (9.42%)
*Staphylococcus haemolyticus*	21 (1.86%)	6 (0.53%)
*Staphylococcus hominis*	43 (3.8%)	27 (2.40%)
*Staphylococcus lentus*	3 (0.26%)	1 (0.08%)
*Staphylococcus lugdunensis*	1 (0.08%)	2 (0.17%)
*Staphylococcus pettenkoferi*	2 (0.17%)	1 (0.08%)
*Staphylococcus saprophyticus*	2 (0.17%)	0
*Staphylococcus schleiferi*	2 (0.17%)	0
*Staphylococcus sciuri*	1 (0.08%)	0
*Staphylococcus simulans*	1 (0.08%)	0
coagulase-negative *Staphylococcus* spp.	4 (0.35%)	2 (0.17%)
*Staphylococcus warneri*	1 (0.08%)	0
*Streptococcus acidominimus*	1 (0.08%)	2 (0.17%)
*Streptococcus agalactiae*	2 (0.17%)	3 (0.51%)
*Streptococcus anginosus*	2 (0.34%)	0
*Streptococcus constellatus*	0	1 (0.08%)
*Streptococcus milleri*	1 (0.08%)	0
*Streptococcus mitis*	1 (0.08%)	1 (0.08%)
*Streptococcus oralis*	1 (0.08%)	0
*Streptococcus parasanguinis*	2 (0.34%)	0
*Streptococcus pneumoniae*	3 (0.51%)	4 (0.35%)
*Streptococcus pyogenes*	1 (0.08%)	1 (0.08%)
*Streptococcus sanguis*	0	1 (0.08%)
*Streptococcus viridans*	16 (1.42%)	14 (1.24%)
*Streptococcus* group C	2 (0.34%)	1 (0.08%)
*Corynebacterium* group JK	2 (0.34%)	2 (0.34%)
*Gemella* spp.	1 (0.08%)	1 (0.08%)
*Lactobacillus* sp.	0	1 (0.08%)
*Leuconostoc* sp.	0	1 (0.08%)
*Pediococcus pentosaceus*	1 (0.08%)	0
*Nocardia* sp.	1 (0.08%)	0
Total % of gram-positive bacteria	369 (28.64%)	260 (26.96%)

n_p_, number of patients; n (%), number of isolates (percentage of isolates in patients).

**Table 6:** Antimicrobial resistance and susceptibility of gram-positive bacteria isolated from blood specimens.

Name of Bacteria		*S. epidermidis*	*S. hominis*	*S. aureus*	MRSA	*S. capitis*	*E. faecalis*	*S. viridans*	*S. haemolyticus*	*E. faecium*
No. of isolates		230	70	55	47	38	36	30	27	21
CF	S %	12.1	24.2	98.2	0	28.9	0	NT	25.9	NT
	R %	87.4	72.8	1.8	100	68.4	100	NT	74.1	NT
CTX	S %	NT	NT	NT	0	NT	0	36.67	NT	NT
	I %	NT	NT	NT	0	NT	0	13.33	NT	NT
	R %	NT	NT	NT	100	NT	100	16.66	NT	NT
CEF	S %	NT	NT	NT	0	NT	0	33.33	NT	NT
	I %	NT	NT	NT	0	NT	0	13.33	NT	NT
	R %	NT	NT	NT	100	NT	100	16.66	NT	NT
CAZ	S %	NT	NT	NT	0	NT	0	NT	NT	NT
	I %	NT	NT	NT	0	NT	0	NT	NT	NT
	R %	NT	NT	NT	100	NT	100	NT	NT	NT
CPE	S %	NT	NT	NT	0	NT	0	40	NT	NT
	I %	NT	NT	NT	0	NT	0	3.33	NT	NT
	R %	NT	NT	NT	100	NT	100	13.33	NT	NT
AMP	S %	NT	NT	NT	0	NT	97.22	36.66	NT	14.28
	I %	NT	NT	NT	0	NT	0	36.66	NT	0
	R %	NT	NT	NT	100	NT	2.8	20	NT	85.7
AM-CLAV	S %	12.1	24.2	96.3	0	28.9	NT	NT	25.9	NT
	R %	87.4	71.4	1.8	100	6.15	NT	NT	74.1	NT
OX	S %	12.1	24.2	98.18	0	28.9	NT	NT	25.9	NT
	R %	87.4	74.2	1.8	100	68.4	NT	NT	74.1	NT
PEN	S %	NT	NT	NT	0	NT	NT	0.4	NT	NT
	I %	NT	NT	NT	0	NT	NT	40	NT	NT
	R %	NT	NT	NT	100	NT	NT	16.6	NT	NT
GM	S %	0.46	NT	NT	2.1	NT	66.6	NT	NT	52.3
	R %	0.93	NT	NT	0	NT	22.2	NT	NT	23.8
CIP	S %	0	0	1.82	0	0	0	NT	0	4.76
	I %	0	0	0	0	0	2.77	NT	0	0
	R %	0.93	1.4	0	2.1	5.2	2.8	NT	3.7	4.7
ERY	S %	13.95	18.57	67.27	72.34	23.68	NT	NT	22.22	NT
	I %	1.4	0	0	0	0	NT	NT	0	NT
	R %	84.1	80	32.7	27.6	71.1	NT	NT	77.7	NT
CC	S %	46.1	92.85	89.1	80.85	55.26	0	NT	66.67	NT
	I %	0.93	0	0	0	0	0	NT	3.7	NT
	R %	57.2	7.1	9.1	19.1	42.1	1	NT	29.6	NT
SXT	S %	43.2	40	94.5	70.2	68.4	NT	NT	66.6	NT
	R %	56.2	57.1	5.5	29.7	26.3	NT	NT	33.3	NT
VA	S %	87.4	72.8	12.7	100	78.9	77.7	66.6	70.3	38.1
	I %	0	0	0	0	0	0	0	0	38.1
	R %	0	0	0	0	0	0	3.3	0	57.1

S %, susceptibility; I %, intermediate; R %, resistance; NT (Not Tested). AM-CLAV, amoxicillin-clavulanate; AMP, ampicillin; CAZ, ceftazidime; CC, clindamycin; CEF, cephalothin; CF, cefalotin; CIP, ciprofloxacin; CPE, ceftolozane-tazobactam; ERY, erythromycin; GM, gentamicin; OX, oxacillin; PEN, penicillin; SXT, trimethoprim-sulfamethoxazole; VA, vancomycin.

**Table 7:** Antimicrobial resistance and susceptibility of gram-negative bacteria isolated from blood specimens.

Name of Bacteria		*K. pneumoniae* (non-ESBL)	*E. coli* (non-ESBL)	*P. aeruginosa*	*E. coli* (ESBL)	*E. cloacae*	*K. pneumoniae* (ESBL)	*A. baumanni*	*S. marcescens*
No. of isolates		130	69	54	39	34	33	23	20
CF	S %	1.54	2.9	NT	0	2.94	0	NT	0
	I %	41.54	7.25	NT	0	0	0	NT	0
	R %	55.4	84.1	NT	100	91.2	100	NT	95
CXM	S %	69.23	72.46	NT	0	14.7	0	NT	0
	I %	1.54	1.45	NT	0	2.94	0	NT	0
	R %	23.8	15.9	NT	100	76.5	100	NT	95
CTX	S %	3.85	14.49	NT	0	58.82	0	NT	70
	I %	0	0	NT	0	2.94	0	NT	5
	R %	23.1	10.1	NT	100	23.5	100	NT	10
CEF	S %	3.1	14.49	NT	0	58.82	0	NT	75
	I %	0	0	NT	0	5.88	0	NT	5
	R %	23.1	10.1	NT	100	23.5	100	NT	10
CAZ	S %	2.3	0	72.22	0	14.7	0	21.73	25
	I %	0	1.44	7.4	0	0	0	4.34	0
	R %	23.1	8.7	20.4	100	8.8	100	73.9	0
CPE	S %	0.77	4.35	12.96	0	2.94	0	4.35	25
	I %	0	1.45	0	0	11.76	0	4.36	0
	R %	23.1	7.2	18.5	100	8.8	100	73.9	0
AMP	S %	1.54	29	1.85	0	0	0	NT	0
	I %	0	0	0	0	0	0	NT	0
	R %	96.2	17.01	0	100	94.1	3.03	NT	100
AM-CLAV	S %	73.1	46.38	NT	0	2.94	0	NT	0
	I %	0	7.7	NT	0	0	0	NT	0
	R %	26.2	23.2	NT	100	91.2	100	NT	100
PEN	S %	NT	NT	NT	0	NT	0	NT	NT
	R %	NT	NT	NT	100	NT	100	NT	NT
TZP	S %	13.85	14.49	66.67	0	50	0	8.7	30
	I %	0	0	25.92	0	5.88	0	4.34	0
	R %	23.8	8.7	7.41	2.5	5.8	6.06	73.9	0.5
GM	S %	86.92	89.86	94.44	51.28	94.11	70	39.13	100
	I %	0	1.45	0	0	0	0	4.35	0
	R %	12.3	7.2	5.56	25.6	5.8	30.3	56.5	0
AN	S %	10	7.25	11.11	33.33	17.65	30.3	17.4	15
	I %	0	0	3.7	0	0	0	0	0
	R %	17.6	1.4	0	0	0	3.03	39.1	0
CIP	S %	3.85	5.8	16.67	5.13	5.88	3.03	17.4	25
	I %	1.54	0	0	0	0	3.03	0	0
	R %	20.7	11.5	9.26	43.5	8.8	12.12	69.5	0
LEVO	S %	2.31	4.35	9.26	5.13	5.88	6.06	17.4	15
	I %	0.7	0	3.7	5.13	0	0	0	0
	R %	20.7	8.7	11.11	41.03	8.8	9.09	69.5	0
IMP	S %	1.54	5.8	7.41	100	17.65	100	17.4	15
	I %	0	0	5.56	0	0	0	0	0
	R %	20.7	2.9	27.78	0	2.9	0	69.5	10
MER	S %	4.62	7.25	9.26	100	20.56	100	17.39	0.05
	I %	0	1.45	0	0	0	0	0	0
	R %	20	1.4	27.7	0	2.9	0	69.5	10
ERT	S %	0.77	5.8	NT	100	14.7	100	NT	0
	R %	19.2	2.9	NT	0	2.9	0	NT	100
AZT	S %	0	1.45	0	NT	0	NT	NT	10
	I %	0	0	3.7	NT	0	NT	NT	0
	R %	18.46	4.3	5.56	NT	2.9	NT	NT	5
CL	S %	NT	0	14.81	NT	NT	NT	21.74	NT
	I %	NT	1.45	0	NT	NT	NT	0	NT
	R %	NT	0	0	NT	NT	NT	39.1	NT
SXT	S %	2.31	10.14	NT	0	0	0	4.35	25
	I %	0	0	NT	0	0	0	0	0
	R %	21.54	2.9	NT	2.5	2.9	3.03	0	0

S %, susceptibility; I %, intermediate; R %, resistance; NT (Not Tested). AM-CLAV, amoxicillin-clavulanate; AMP, ampicillin; AN, amikacin; AZT, aztreonam; CAZ, ceftazidime; CEF, cephalothin; CF, cefalotin; CIP, ciprofloxacin; CL, chloramphenicol; CPE, ceftolozane-tazobactam; CTX, cefotaxime; CXM, cefuroxime; ERT, ertapenem; IMP, imipenem; GM, gentamicin; LEVO, levofloxacin; MER, meropenem; PEN, penicillin; SXT, trimethoprim-sulfamethoxazole; TZP, piperacillin-tazobactam.

## Data Availability

All data related to this investigation has been included in the manuscript.
